# Inhomogeneous Broadening of Photoluminescence Spectra and Kinetics of Nanometer-Thick (Phenethylammonium)_2_PbI_4_ Perovskite Thin Films: Implications for Optoelectronics

**DOI:** 10.1021/acsanm.1c00984

**Published:** 2021-06-16

**Authors:** Vladimir S. Chirvony, Isaac Suárez, Jesús Rodríguez-Romero, Rubén Vázquez-Cárdenas, Jesus Sanchez-Diaz, Alejandro Molina-Sánchez, Eva M. Barea, Iván Mora-Seró, Juan P. Martínez-Pastor

**Affiliations:** † UMDO, Instituto de Ciencia de los Materiales, Universidad de Valencia, Paterna, Valencia 46980, Spain; ‡ Escuela Técnica Superior de Ingeniería, Universidad de Valencia, Burjassot, Valencia 46100, Spain; § Institute of Advanced Materials (INAM), Universitat Jaume I, Castelló de la Plana, Castelló 12006, Spain; ∥ Facultad de Química, Universidad Nacional Autónoma de México, Coyoacán, Ciudad de México 04510, Mexico; ⊥ Facultad de Ciencias Químicas, Universidad de Colima, Colima 28400, Mexico

**Keywords:** 2D halide perovskite, polycrystalline film, excitonic photoluminescence, absorption, inhomogeneous broadening

## Abstract

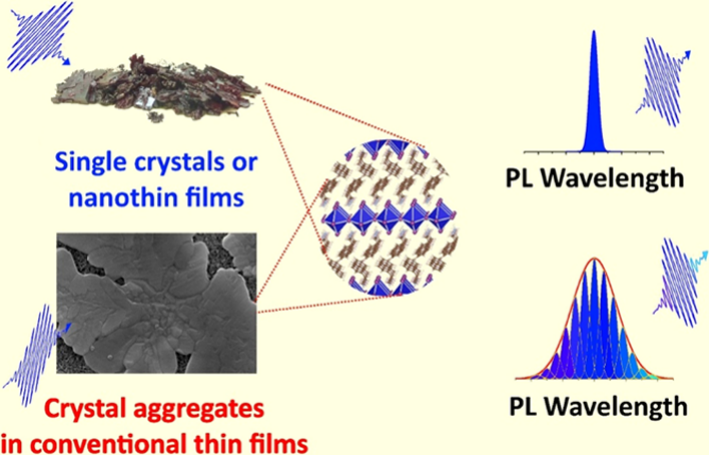

An outstanding potentiality of layered two-dimensional (2D) organic–inorganic hybrid perovskites (2DHPs) is in the development of solar cells, photodetectors, and light-emitting diodes. In 2DHPs, an exciton is localized in an atomically thin lead(II) halide inorganic layer of sub-nanometer thickness as in a quantum well sandwiched between organic layers as energetic and dielectric barriers. In previous years, versatile optical characterization of 2DHPs has been carried out mainly for thin flakes of single crystals and ultrathin (of the order of 20 nm) polycrystalline films, whereas there is a lack of optical characterization of thick (hundreds of nanometers) polycrystalline films, fundamentals for fabrication of devices. Here, with the use of photoluminescence (PL) and absorption spectroscopies, we studied the exciton behavior in ∼200 nm polycrystalline thin films of 2D perovskite (PEA)_2_PbI_4_, where PEA is phenethylammonium. Contrary to the case of ultrathin films, we have found that peak energies and line width of the excitonic bands in our films demonstrate unusual extremely weak sensitivity to temperature in 20–300 K diapason. The excitonic PL band is characterized by a significant (∼30 meV) Stokes shift with respect to the corresponding absorption band as well as by a full absence of the exciton fine structure at cryogenic temperatures. We suggest that the observed effects are due to the large inhomogeneous broadening of the excitonic PL and absorption bands resulting from the (PEA)_2_PbI_4_ band gap energy dependence on the number of lead(II) halide layers of individual crystallites. The characteristic time of the exciton energy funneling from higher- to lower-energy crystallites within (PEA)_2_PbI_4_ polycrystalline thin films is about 100 ps.

## Introduction

Ruddlesden–Popper two-dimensional (2D) organic–inorganic hybrid perovskites (2DHPs) having the formula A_2_MX_4_ are crystalline structures of alternating inorganic monolayers and organic bilayers which consist of stacks of octahedral metal–halide (M–X) monolayers (M is typically a divalent metal and X a halide anion) each separated by bulky organo-ammonium cations (A) to maintain charge balance and the layered structure.^1−6^ Very recently, the class of Ruddlesden–Popper 2D halide perovskites has been extended by all-inorganic Cs_2_PbX_4_ compounds possessing optoelectronic response.^7−9^ From the physical point of view, 2DHPs belong to quantum well superlattices, in which strong quantum and dielectric confinement effects^2^ create stable room temperature excitons with binding energies >150 meV in the M–X layers.^10^ Besides, it is shown in some works that 2D A_2_PbI_4_ films exhibit multiple narrow resonances in excitonic absorption and photoluminescence (PL) spectra at cryogenic temperatures.^2,11,12^ It was initially suggested that these resonances were belonging to the vibronic structure of the material, which is caused by coupling of excitons in the inorganic framework with phonons in the organic cations,^11,13,14^ but later other explanations related to polaron–exciton interactions were proposed.^12,15^ In addition to the aforementioned fine structure of exciton bands at low temperatures, a strong temperature dependence of the exciton transition energy, a significant broadening of the exciton band in absorption and emission, and a small Stokes shift of the PL band relative to the absorption band^11,16−23^ were found for 2DHPs. To this extent, the understanding of the photophysical properties of 2DHPs is a must in order to completely develop the full potential of these materials for optoelectronic applications.

In this context, an analysis of the literature shows that until now, the optical properties of excitons have been investigated mainly for single crystals in the form of flakes exfoliated from bulk single crystals^16−21^ or for very thin (about 20 nm) polycrystalline films prepared by spin-coating deposition of extremely low-concentrated stoichiometric solutions of precursors.^11,22,23^ Meanwhile, for use in photovoltaic and optoelectronic devices, 2DHP layers with thicknesses of the order of 100 nm and more are required.^24−26^ However, for these systems that constitute likely the most important type of 2DHP films from the point of view of applications, the behavior of excitons has not been investigated. In this sense, it is important to recognize that the polycrystalline nature observed in these films prepared by fast solution deposition methods is largely determined by the specific conditions of synthesis during the spin-coating process that is going to determine significantly the final properties of the film. The methodological approach (specific concentrations, solvent viscosity, spin parameters, the application of high temperatures, antisolvent, etc.) used in course of fabrication of high-quality thin films has important influence on the film parameters such as film thickness and substrate coating by the film. Besides, very specific features, such as grain size distribution, grain boundaries, and crystallinity, are also affected by the method of fabrication. For example, when a very diluted precursor solution is used, the obtained film is usually very thin.^21^ On the other hand, it is well known that the increase of the annealing temperature of spin-coated thin films leads to both bigger grain sizes and thicker films^27^ owing to the modification of the rate of nucleation growth, influencing the size and distribution of micro-crystallites throughout the film.^28,29^ As far as we know, the behavior of excitons in such polycrystalline thin films has not been sufficiently studied, although the solution method for the preparation of 2DHPs is very popular in the current studies of optoelectronic devices, such as perovskite light-emitting diodes^26^ and solar cells.^25^

**Scheme 1:**
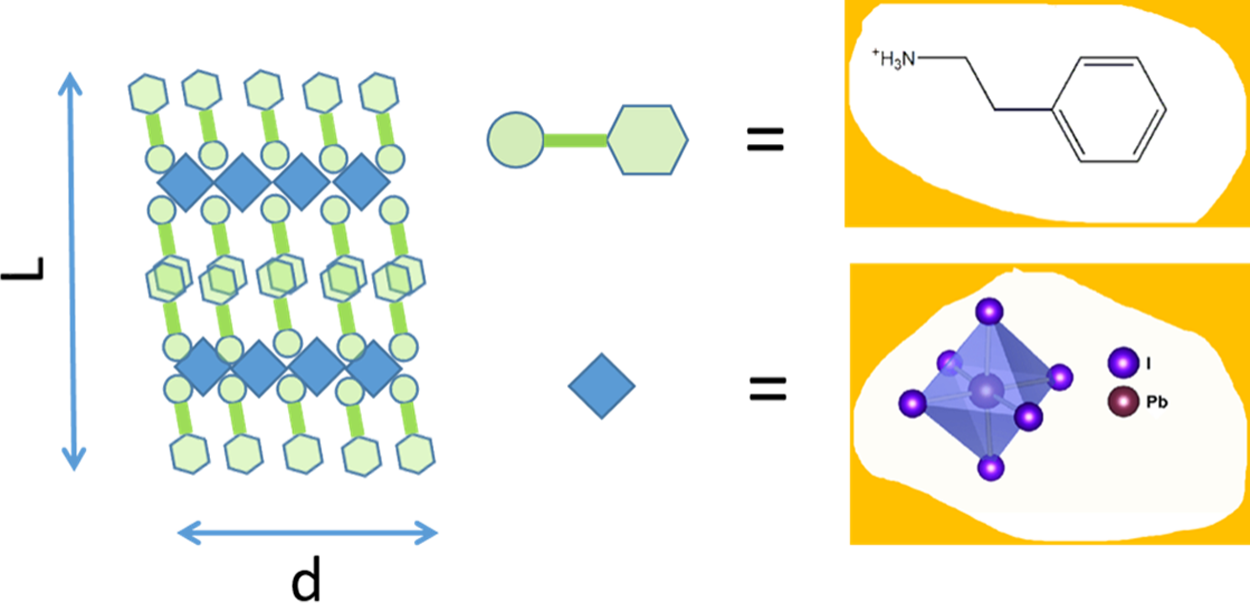
Structure of (PEA)_2_PbI_4_ Perovskite; *L* is the Number of Inorganic Sheets in an Individual Crystallite (*L* = 2 in the Figure) and *d* is the Transverse Dimension of a Crystallite

In the present work, we fill this gap and investigate the absorption and PL properties of polycrystalline films of (PEA)_2_PbI_4_ 2D perovskite, where PEA is phenethylammonium (see Scheme 1), in a wide interval of temperatures, 20–300 K. We found that (PEA)_2_PbI_4_ polycrystalline films exhibit for the excitonic band, in both absorption and PL, a full absence of the exciton fine structure, even at cryogenic temperatures. Additionally, we determine a unique extremely weak sensitivity of the exciton spectral parameters to temperature, as the PL peak energy or the full width at half-maximum (fwhm). We hypothesize that the observed unusual effects are due to the large inhomogeneous broadening of the PL excitonic band. The origin of the spectral inhomogeneity is discussed in terms of the structural heterogeneity of nominally equal (PEA)_2_PbI_4_ nano- and micro-structures forming the films. The knowledge of the fundamental physical properties of this material in thin-film configuration will help in the application of this system as the basis for the development of optoelectronic devices.

## Results and Discussion

In the present work, we investigate (PEA)_2_PbI_4_ perovskite films, which were fabricated by a modified hot casting method; for details, see the Supporting Information, Experimental Section. For this, phenethylammonium iodide and PbI_2_ were mixed in the corresponding stoichiometry and dissolved in a dimethylformamide (DMF)/dimethyl sulfoxide (DMSO) mixture (1:0.095). The perovskite precursor solution was heated at 70 °C during all the process. Subsequently, the perovskite precursor solution was spin-coated on glass substrates in a one step process. Finally, the samples were annealed at 100 °C for 10 min. On the basis of the film optical density in the excitonic band, the film thickness was evaluated to be about 200 nm.

**Figure 1:**
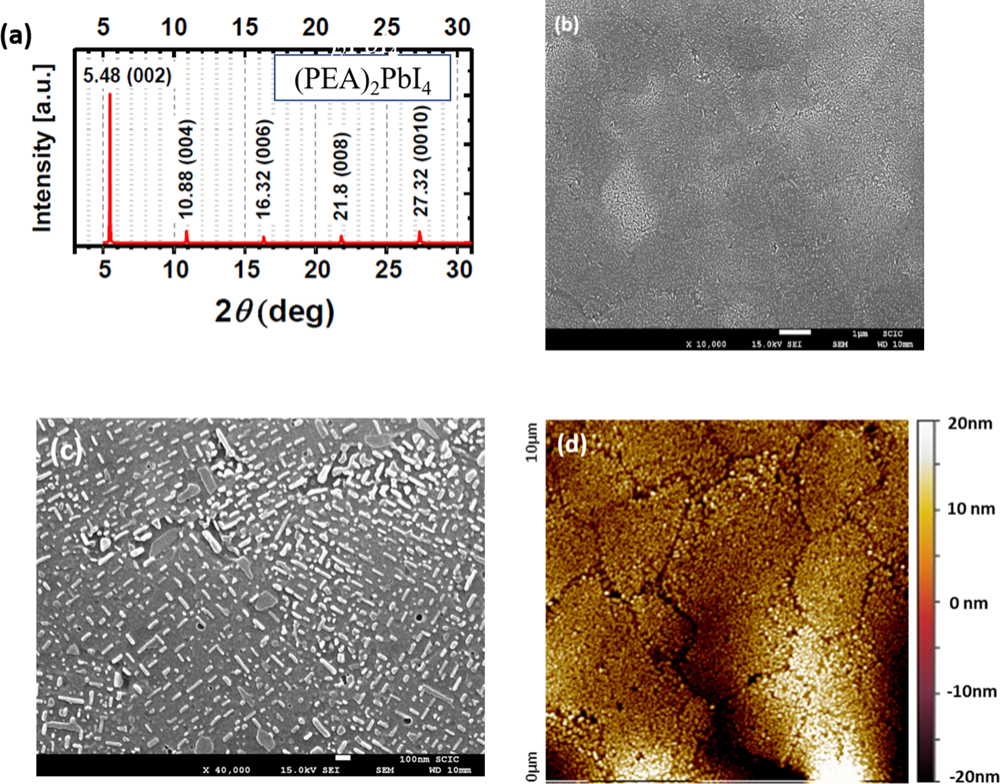
(a) XRD pattern; SEM (scale bar 1 μm in (b) and 100 nm in (c)) and (d) AFM images of (PEA)_2_PbI_4_ perovskite films.

To characterize the crystal structure and a potential preferential orientation of grains in polycrystalline thin films, X-ray diffraction (XRD) measurements were performed. Figure 1a shows the XRD pattern of a (PEA)_2_PbI_4_ thin film spin-coated from DMF/DMSO solution. The presence or absence of certain diffraction peaks in the XRD patterns of thin films recorded in the Bragg–Brentano scanning mode can give first indications for potentially preferred crystal orientation within the polycrystalline samples. Furthermore, the 2θ position of the diffraction peaks corresponding to the stacking direction of the perovskite interlayers reveals the dimensions of the unit cell and therefore the number of octahedra layers, value known as *n*. Our thin-film (PEA)_2_PbI_4_ samples exhibit pronounced peaks at the diffraction angles 2θ = 5.48, 10.88, 16.32, 21.8, and 27.32°, which can be indexed as the (002), (004), (006), (008), and (0010) reflections of (PEA)_2_PbI_4_. XRD line broadening allowed us to evaluate the crystallite size, which is found to be between 60 and 120 nm, see Note 1 and Table S1 in the Supporting Information.

The scanning electron microscopy (SEM) (Figure 1b,c) and atomic force microscopy (AFM) (Figure 1d) images of (PEA)_2_PbI_4_ thin films demonstrate that they consist of crystals of several microns in size closely adjacent to each other and covered by elongated structures up to 100–200 nm in length. On the basis of the AFM image, we determined the roughness profile of the film surface, see Figure S2, and calculated the root-mean-square value to be 1.68 nm, showing that the roughness of the film is rather small.

**Figure 2:**
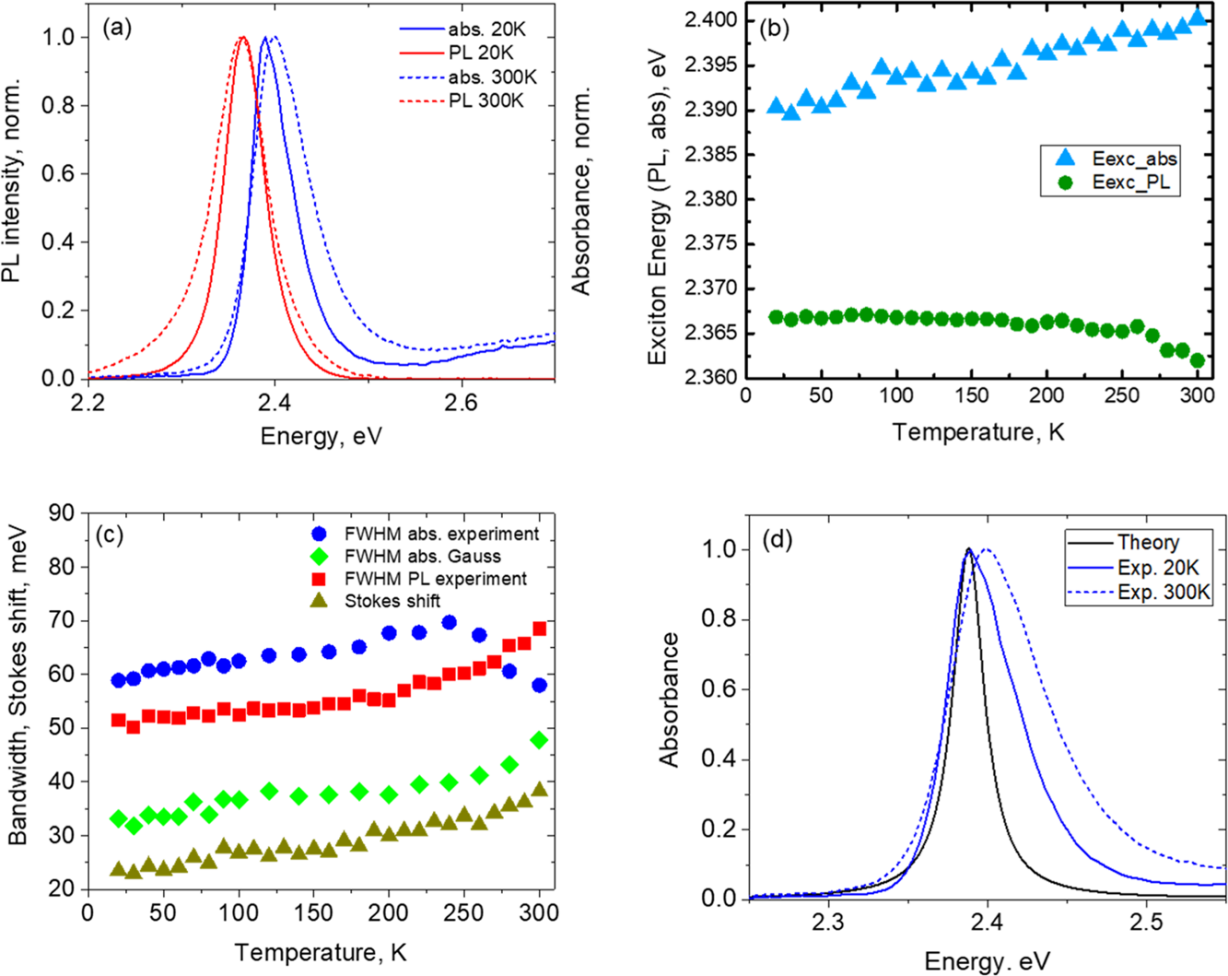
Spectral characteristics of the investigated (PEA)_2_PbI_4_ polycrystalline films as a function of temperature: (a) normalized absorption and PL spectra recorded at 20 and 300 K; (b) exciton energies measured as peak maxima in absorption and PL; and (c) fwhm of the experimental excitonic bands in absorption and PL, fwhm of a Gauss profile modeling the exciton absorption band, see the Supporting Information for details, and a Stokes shift between the maxima of the experimental absorption and PL excitonic bands are also shown. (d) Absorption spectrum obtained by *ab initio* calculations, black curve (see the text for details), as well as experimental absorption spectra recorded at 20 and 300 K.

Figure 2a shows the absorption and PL spectra of our (PEA)_2_PbI_4_ films recorded at 20 and 300 K. An important feature of the presented spectra is that both the position of the exciton energy, considered from the spectra maxima, and their corresponding fwhm are weakly dependent on temperature, see Figure 2b,c. Indeed, the position of the maximum of the exciton absorption band changes by only 10 meV, from 2.39 to 2.40 eV, with increasing temperature from 20 to 300 K, see Figure 2b.

In this way, the position of the PL band maximum remains almost unchanged in the range from 20 to 160 K, being at the level of 2.367 eV, and then the exciton energy decreases by only 5 meV to 2.362 eV at 300 K, see Figure 2b. This leads to an increase in the Stokes shift between the exciton energies in absorption and PL from 23 to 38 meV, when temperature increases from 20 to 300 K, see Figure 2c. When the temperature changes in such a large range, the fwhm of the excitonic contour, both in absorption and in emission, alters insignificantly, see Figure 2c. Indeed, the fwhm of the Gaussian contour modeling the absorption band slightly increases from 32 to 40 meV with increasing temperature from 20 to 240 K and then grows more sharply to 48 meV at 300 K, see Figure 2c. A description of the absorption band modeling by a Gaussian contour is presented in Figure S3 caption.

For modeling optical properties of (PEA)_2_PbI_4_ and confirm the excitonic origin of the detected resonances, we used the *ab initio* calculation method, see the Supporting Information and ref (30) for further details. Figure 2d shows the calculated absorption spectrum of (PEA)_2_PbI_4_ in comparison with the experimental spectra recorded at 20 and 300 K. As one can see, the position of the band maximum in the calculated spectrum practically coincides with that in the experimental spectrum detected at 20 K.

**Figure 3:**
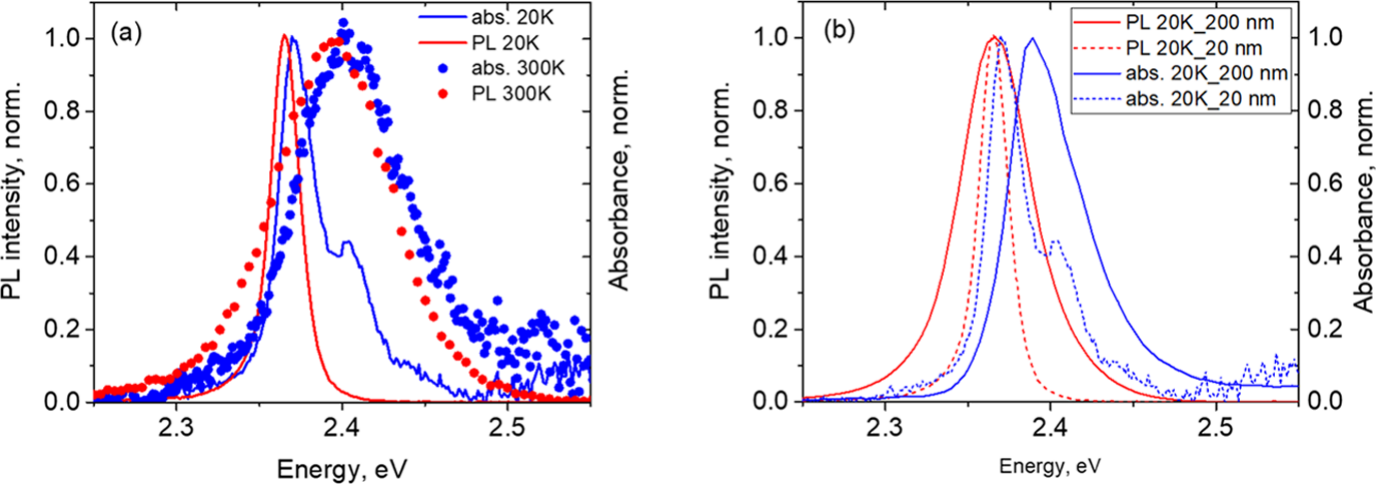
(a) Normalized absorption and PL spectra of 20 nm (PEA)_2_PbI_4_ films prepared following ref (11) and recorded at 20 and 300 K and (b) comparison of PL and absorption spectra recorded at 20 K for our 200 nm samples with 20 nm samples prepared following ref (11).

It is worth to note here that, contrary to what we observe, in the case of previously studied exfoliated single crystals^16^ or very thin polycrystalline^22^ 2DHPs, a strong dependence of the exciton energies and spectral widths of absorption and PL excitonic bands on temperature, as well as a fine structure of excitonic bands at cryogenic temperatures, were reported. In order to understand the reason that promotes the different optical behavior exhibiting our rather thick (about 200 nm) polycrystalline (PEA)_2_PbI_4_ films as compared to previously studied very thin polycrystalline samples in other laboratories, we completely reproduced the method of fabrication of (PEA)_2_PbI_4_ films used in ref (11). The samples prepared with this alternative method^11^ possess a film thickness of about 20 nm on the basis of the film optical density in the excitonic band. The absorption and PL spectra recorded for this 20 nm polycrystalline (PEA)_2_PbI_4_ film at room and low temperatures are shown in Figure 3a. As one can see, much stronger temperature-induced changes of the exciton energies in the absorption (2.3706 eV at 20 K to 2.4052 eV at 300 K) and PL (2.3650 eV at 20 K to 2.3941 eV at 300 K) spectra, as well as significant temperature broadening of the excitonic bands in PL, from 21 to 82 meV, and in absorption, from 27 to 77 meV, in the 20 to 300 K diapason are found for this 20 nm (PEA)_2_PbI_4_ film, see Figure 3, as compared to the above described 200 nm thick sample, see Figure 2.

Thus, the above comparison clearly shows that the 20 nm (PEA)_2_PbI_4_ films exhibit a fine structure that is typical for single-crystalline samples and ultrathin polycrystalline films,^11,22,23^ while it is lost for 200 nm polycrystalline films prepared by the fast spin-coating of a stoichiometric solution of precursors. The only reasonable explanation for such a spectral behavior of the latter may be the suggestion that the structural units (grains) constituting the polycrystalline layers have slightly different exciton energies that results in the broadening of the excitonic band. Therefore, we suggest that our 200 nm polycrystalline films are spectrally inhomogeneous, and the corresponding absorption and PL bands are inhomogeneously broadened. Indeed, the fwhm of the absorption and PL bands at 20 K of the 200 nm thick films are 2–2.5 times larger than those of the 20 nm sample, as demonstrated by the comparison shown in Figure 3b.

In order to explore the reason for the important inhomogeneous broadening of the excitonic bands in the studied 200 nm polycrystalline (PEA)_2_PbI_4_ films, the microstructure of the films was investigated by SEM and AFM images, see Figure 1b–d. However, the observed microstructure, with flat crystals of several microns in lateral size covered by much smaller elongated structures up to 100–200 nm in length, is very similar to the literature microstructural data published for thinner polycrystalline 2DHP films.^21^ Beyond the microstructure of the film, several other mechanisms can be responsible for the inhomogeneous broadening of the studied polycrystalline (PEA)_2_PbI_4_ films:

(i) Dependence of the spectral position of the PL spectra on the orientation and polarization of the exciting light relative to the plane of the 2D structure.^31^ Nevertheless, for realization of this inhomogeneous broadening mechanism, it is necessary that the orientation of individual sub-micro- and micro-microcrystals in the film is random to detect PL not only from the film plane but also from edges. However, since XRD data indicate that the individual crystals are oriented primarily parallel to the substrate plane, see Figure 1a, we consider the participation of this effect in inhomogeneous broadening to be unlikely.
(ii) Dependence of the position of the PL spectra on the concentration of defect states on the surface: the higher the concentration of emitting shallow defects, the more the PL spectrum is shifted relative to the defect-free excitonic transition.^32,33^ Nonetheless, we believe that this effect cannot be the main mechanism for the onset of inhomogeneous broadening since it leads to inhomogeneity only in the PL spectra, while our results point to inhomogeneous broadening both in the emission and absorption bands.
(iii) Emission of the magnetic dipole exciton, which is red-shifted in comparison to the standard electric dipole emission.^34^ In our case, this effect can hardly lead to inhomogeneous broadening of the excitonic band since this requires, in view of the orthogonal orientation of the magnetic dipole relative to the electric one, that the orientation of the individual crystals in the layer is random or orthogonal to the surface.
(iv) Dependence of the optical band gap on the individual crystals lateral size. Indeed, it has been recently found that ultrathin single-layer colloidal (PEA)_2_PbI_4_ nanosheets exhibit rather strong dependence of the excitonic band energy on the lateral size: a considerable short-wavelength shift of about 9 nm from 525 to 516 nm (or ≈40 meV) of the PL excitonic band is found when the nanosheet lateral size decreases from about 530–100 nm.^35^ However, it remains unclear what is the mechanism of the found dependence of the nanosheet band gap on its lateral size at the conditions when the size is much larger than the excitonic Bohr radius (<10 nm). We do not exclude that the effect discovered by Yang et al.^35^ is due to the dependence of the optical band gap on the uncontrollable nanosheet thickness (see the next item).
(v) Dependence of the optical band gap on crystallite thickness. As shown in refs (21) and (36), the optical gap in (PEA)_2_PbI_4_ single-crystalline sheets is dependent on the thickness of the sheets, that is, on the number *L* of (PEA)_2_PbI_4_ monolayers, due to the thickness-dependent reduction of the dielectric screening effect, in comparison to crystallites with a large number of monolayers possessing a bulk like behavior. In this case, the emission peak energy difference between a monolayer, *L* = 1, and bulk samples, *L* = ∞, is about 35 meV, from 518 to 526 nm, respectively,^21^ as also measured by us using exfoliated nanosheets (not shown here) of a (PEA)_2_PbI_4_ single crystal. The described observations suggest that this mechanism is the most probable one being responsible for the inhomogeneous broadening of excitonic bands in the investigated 200 nm polycrystalline (PEA)_2_PbI_4_ films consisting of many individual crystallites with a broad *L*-distribution.

We hypothesize therefore that, in rather thick polycrystalline films prepared by spin-coating of concentrated precursor solutions, there is a distribution of nano- and micro-sized crystallite structures with its subsequent exciton energy distribution. In this case, the difference between the exciton maximum and minimum energies within the distribution exceeds the temperature broadening of the exciton band of individual 2D (PEA)_2_PbI_4_ crystallites. Thus, one would expect the spectral width of the exciton band to be weakly dependent on temperature, especially in absorption. As for the PL spectra, we suggest that there should be a fast transfer of excitation energy from higher to lower energy band gaps, that is, from thinner to thicker individual crystallites. As a result of such fast energy funneling process, faster than PL lifetime, a majority of the PL photons will be emitted mainly by individual crystallites with the lowest exciton energy. This should lead to a significant Stokes shift of the PL at any temperature, as it is observed experimentally, see Figure 2c, and consistent with strong exciton localization effects in (PEA)_2_PbI_4_ crystallites as it is well known for quantum wells,^37^ in contrast to free or weakly localized excitons in thermal equilibrium.^38^

**Figure 4:**
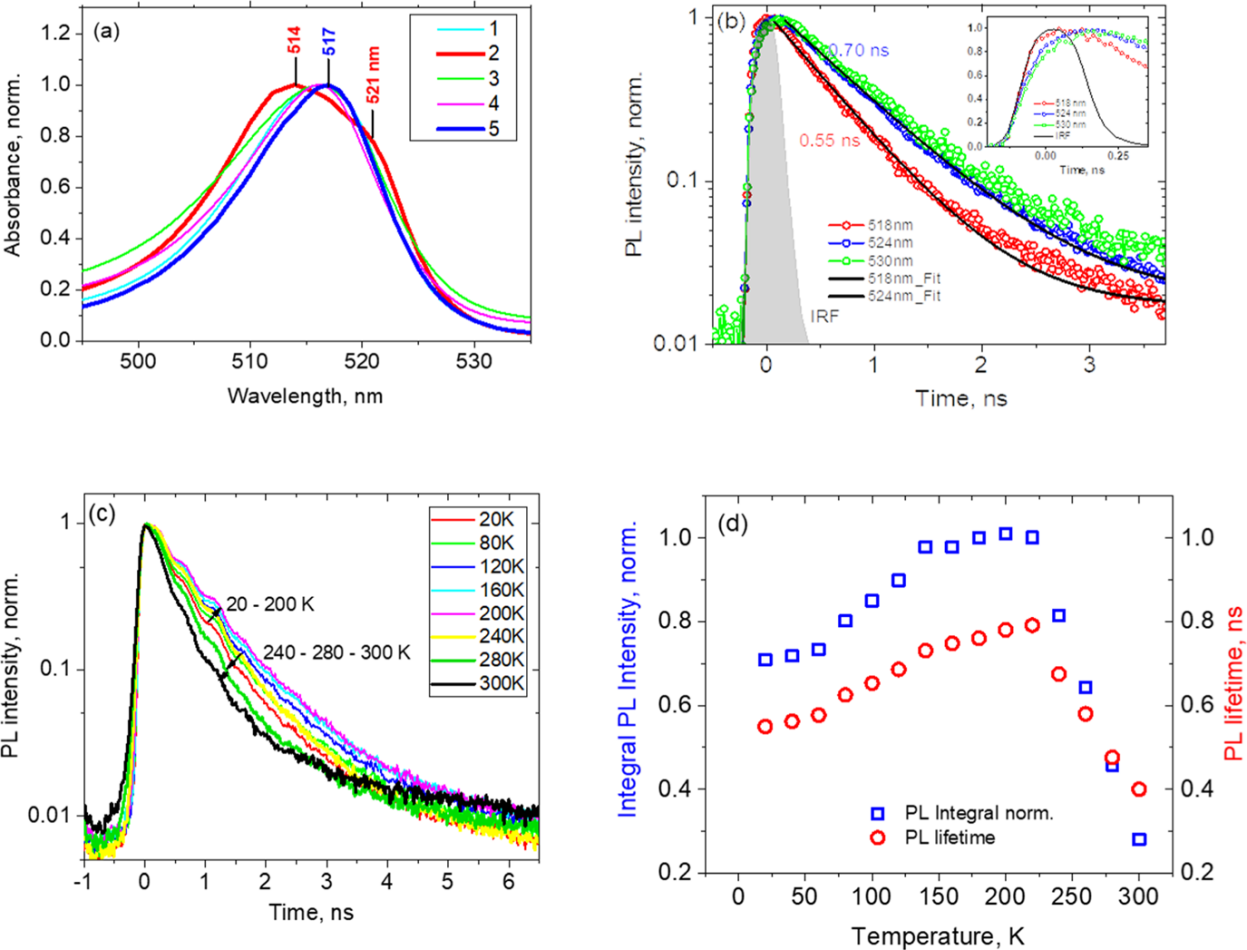
Absorption and PL transient properties of 200 nm polycrystalline (PEA)_2_PbI_4_ films: (a) excitonic absorption band measured for films fabricated in different syntheses (the most dissimilar spectra 2 and 5 are shown by thicker lines, and the wavelengths of their maxima and a shoulder are also indicated); (b) PL decay kinetics detected at different wavelengths (the inset shows the same at a shorter time scale); (c) decay kinetics of the full PL spectrum at different temperatures; and (d) dependence of the PL integral intensity and lifetime on temperature.

A careful analysis of optical data measured for our 200 nm (PEA)_2_PbI_4_ polycrystalline films allows one to find numerous lines of evidence of inhomogeneous broadening of the excitonic band in absorption and emission. First, it turned out that the contour of the excitonic absorption band has a complex shape and varies slightly from synthesis to synthesis, see Figure 4a, which we interpret as a result of changes in the distribution of the number of crystallites with different *L* forming the film. Second, we also observe the dependence of the PL decay kinetics on the detection wavelength which we interpret as a result of the excitation energy funneling within a set of individual crystallites with slightly different exciton energies, Figure 4b. Indeed, the PL transients detected at longer wavelengths, that is, lower exciton energies, demonstrate a rise intensity part which results in a delay for reaching of the intensity maximum. It means that the PL spectrum is inhomogeneously broadened and energy funneling from higher to lower energy individual structures requires some time on a subnanosecond scale, see the inset in Figure 4b, observing that the time constant of the delayed PL growth is about 100 ps. At the same time, the PL decay kinetics are well fitted by monoexponential functions with similar decay times in the interval of 0.55–0.70 ns at different wavelengths within the PL band. Thus, the characteristic time of the excitation energy funneling from higher- to lower-energy individual crystallites in 200 nm polycrystalline (PEA)_2_PbI_4_ films is about 100 ps, which qualitatively agrees with the times of energy funneling between phases with different n’s in similar quasi-2D perovskites known from the literature.^39^

Thus, it becomes clear that the PL decay kinetics of the investigated polycrystalline layers measured at any selected wavelength carry information not only about the exciton recombination rate but also about the rate of the PL spectrum long-wavelength shift, that is, spectral diffusion, during the PL decay. Therefore, to obtain objective information about the exciton recombination time, we have to separate this process from the exciton spectral diffusion. To achieve this, we measured the PL decay kinetics for the full PL spectrum. In contrast to the kinetics measured at individual wavelengths, the time dependence of the total integrated intensity of the spectrum does not show any delay of the intensity maximum with respect to the instrument response function, see Figure 4c, which indicates the absence of the spectral diffusion contribution.

Figure 4d shows the integral PL decay kinetics measured at different temperatures in the interval of 20–300 K. There is about 30% increase of the PL lifetime, which is in fact the exciton recombination time, from 0.55 to 0.75 ns when the temperature grows from 20 to 200 K, and then the lifetime shortens abruptly. A similar temperature dependence is observed for the PL integral intensity, see Figure 4d, which is analogous to the dependence found earlier for single-crystalline (PEA)_2_PbI_4_.^1^ Assuming the Arrhenius dependence of the PL integral intensity on temperature in the range of 220–300 K, we obtained the activation energy *E*_a_ of about 250 meV for the temperature-induced PL quenching process, which is in good agreement with the 230 meV exciton binding energy known for single-crystalline (PEA)_2_PbI_4_ samples.^40^ See Figure S4 for more details. Thus, the observed PL quenching at temperatures above 200 K can be well explained by the exciton dissociation.

## Conclusions

In summary, the optical properties of polycrystalline (PEA)_2_PbI_4_ thin films of about 200 nm thickness prepared by one-step spin-coating of stoichiometric solution of precursors are studied in the 20–300 K temperature diapason. At cryogenic temperatures, the excitonic absorption and the PL bands of the films are about 3 times broadened as compared to the analogous ultrathin (20 nm) films. Besides, very weak sensitivity of the exciton energy, spectral width and Stokes shift to temperature, and a full absence of the exciton fine structure at cryogenic temperatures are found. The observed effects are described as a result of an inhomogeneous broadening of the excitonic band due to a broad distribution of crystallites with different thickness *L* that forms the film. Understanding of the properties of thin layers produced with this material is a key aspect in order to develop efficient optoelectronic and photovoltaic devices.

## Supporting Information Available

Experimental section, crystallite size evaluation by the Scherrer formula, surface roughness AFM profile, Gauss contour modeling, and exciton binding energy determination (PDF)
