# Supplementary material for: Inhomogeneous Broadening of Photoluminescence Spectra and Kinetics of Nanometer-Thick (Phenethylammonium)2PbI4 Perovskite Thin Films: Implications for Optoelectronics
Source: ACS Appl Nano Mater. 2021 Jun 16;4(6):6170–7. doi: 10.1021/acsanm.1c00984 (PMC9185684; doi:10.1021/acsanm.1c00984)
Supplement: Supplementary file 1 — an1c00984_si_001.pdf [file an1c00984_si_001.pdf]

SUPPORTING INFORMATION

Inhomogeneous Broadening of Photoluminescence Spectra and Kinetics of Nanometer-Thick (Phenethylammonium)$_2$PbI$_4$ Perovskite Thin Films: Implications for Optoelectronics

Vladimir S. Chirvony[a]*, Isaac Suárez[b], Jesús Rodríguez-Romero[c,d], Rubén Vázquez-Cárdenas[c,e], Jesus Sanchez-Diaz[c], Alejandro Molina-Sánchez[a], Eva M. Barea[c], Iván Mora-Seró[c], and Juan P. Martínez-Pastor[a]*

[a] UMDO, Instituto de Ciencia de los Materiales, Universidad de Valencia, Paterna, Valencia, 46980, Spain

[b] Escuela Técnica Superior de Ingeniería, Universidad de Valencia, Burjassot, Valencia, 46100, Spain

[c] Institute of Advanced Materials (INAM), Universitat Jaume I, Castelló, 12006, Spain

[d] Facultad de Química, Universidad Nacional Autónoma de México, Coyoacán, Ciudad de México, 04510, México

[e] Facultad de Ciencias Químicas, Universidad de Colima, Colima, 28400, México

*e-mails: vladimir.chyrvony@uv.es; martinep@uv.es

Experimental Section

Materials. PbI_2 (99.999%) was purchased from TCI. Phenylethylamine (PEA), HI, diisopropoxide bis(acac) solution (75% in 2-propanol), ethanol, dimethylsulfoxide (DMSO) and dimethylformamide (DMF) were purchased from Sigma-Aldrich. All reagents were used without further purification.

Synthesis of phenylethylammonium iodide (PEAI). 10 mL of phenylethylamine was added in a round bottom flask (100 mL) and then cooled down in an ice bath for 10 min. After that, 10 mL of HI (55% wt aqueous solution) was dropped very slowly. The reaction mixture was vigorously stirred approximately 60 min until a white solid precipitate. The solid was filtered and washed twice with cold diethyl ether. The solid was dried at 70 °C in a vacuum oven for 12 h.

Fabrication of polycrystalline thin films. The substrates (borosilicate glass) were exposed to UV-O_3 during 10 min. A compact layer of TiO_2 was deposited on the substrates by spray pyrolysis of titanium diisopropoxide bis(acac) solution (75% in 2-propanol, Sigma-Aldrich) diluted with absolute ethanol in 1:9 v/v proportion, respectively. The $(PEA)_2PbI_4$ films were fabricated by modified hot casting method with 1:2 PbI_2 (0.1136 g) to PEAI (0.1227 g) molar ratio, 1 mL of DMF and 0.095 mL of DMSO. The perovskite precursor solution (50 μL), which was heated at 70 °C during all the process, was dripped on the substrate and spin coated at 4000 rpm during 20 s. Finally, the samples were annealed at 100 °C during 10 min. The resulting film thickness was about 250-300 nm.

Fabrication of monocrystalline films. Such high-quality ultra-thin films were prepared in two steps following the procedure described in literature [1]. First, $(PEA)_2PbI_4$ crystals are synthesized performing a liquid-liquid extraction of 9 mL of hydriodic acid (57% w/w in water, unstabilized,

Sigma-Aldrich) with a 10% v/v solution of tributyl phosphate (99+%, Acros Organics) in chloroform (Fisher, ACS grade) until the aqueous phase is clear. The HI is placed in a round bottom flask containing 0.498g PEAI and 0.461 g lead (II) iodide (99.999%, Sigma-Aldrich). The flask is heated under nitrogen in a boiling water bath until the solids dissolve. The solution is slowly cooled over 3 h to room temperature and then placed in a refrigerator overnight. The reaction mixture is vacuum filtered and the PEALI crystals are washed with hydriodic acid purified by extraction and then dried under vacuum.

Second, $(PEA)_2PbI_4$ thin films are cast by dissolving the synthesized perovskite crystals in anhydrous acetonitrile (Acros Organics) at a concentration of 15 mg/mL in a nitrogen glove box. The mixture is sonicated until the crystals dissolve. The solution is filtered through a 0.2 μm PTFE syringe filter (Pall Corporation) and spun in a nitrogen glove box by ramping the spin rate over 2 s to 2500 rpm and spinning for 10 s. The $(PEA)_2PbI_4$ thin films are deposited on glass substrates preliminary exposed to UV-O_3 during 10 min, rendering the surface hydrophilic. After spin-casting, the films are annealed for 10 min at 100˚C in the glove box.

Materials Characterization. X-ray diffraction (XRD) measurements of the thin films were taken by an X-ray diffractometer (D8 Advance, Bruker-AXS) (Cu Kα, wavelength λ = 1.5406 Å). Scanning electron microscopy (SEM) images were obtained using a field-emission scanning electron microscope (FEG-SEM JEOL 3100F) operating at 5 kV. Atomic Force Microscopy (AFM) images were obtained using ResiScope mode AFM (Concept Scientific Instrument) with a platinum coated tip. PL, time-resolved PL (TRPL) and absorption measurements of thin films were realized in a closed-cycle He cryostat, which can be cooled down to 15 K. The sample were excited by a 200 fs pulsed Ti:sapphire (Coherent Mira 900D) at a repetition rate of 76 MHz doubled to 405 nm with a BBO crystal. The backscattered PL signal was dispersed by a double 0.3-m focal

length grating spectrograph/spectrometer (1200 g/mm with 750 nm blaze) and detected by a Si micro photon device (MPD) and single-photon avalanche diode (SPAD) photodetector (connected through a multimode optical fiber to the monochromator); the SPAD was attached to a time correlated single photon counting electronic board (TCC900 from Edinburgh Instruments). The instrument response function is about 50 ps. For absorption spectra measurements, a xenon lamp was used.

***Ab initio* calculations.** The *ab initio* calculations of the optical spectra of $(C_6H_5C_2H_4NH_3)_2PbI_4$ perosvkite have been performed using many-body perturbation theory as implemented in the Yambo code [2]. We have worked in the framework of the GW method and the Bethe-Salpeter Equation (BSE). In order to obtain the excitonic states and the optical spectra using BSE, we have calculated the eigenvalues and eigenvectors of the system using density-functional theory (DFT) within the local-density approximation (LDA) as implemented in Quantum Espresso [3]. We have used norm-conserving pseudopotentials, with a basis-set cutoff energy of 90 Ry and a sampling of the Brillouin zone with a 10x10x1 k-grid. In the case of the BSE calculations, we have used a 30x30x1 k-grid, 100 bands for the calculation of the static dielectric screening and the Coulomb cutoff to avoid artificial interaction between replicas of the supercell (see also Fig. S1 for explanation). Note that only spectral position of the excitonic transition was calculated, whereas the Lorentz contour with FWHM of 12 meV was used to present the absorption band in Fig. 2d.

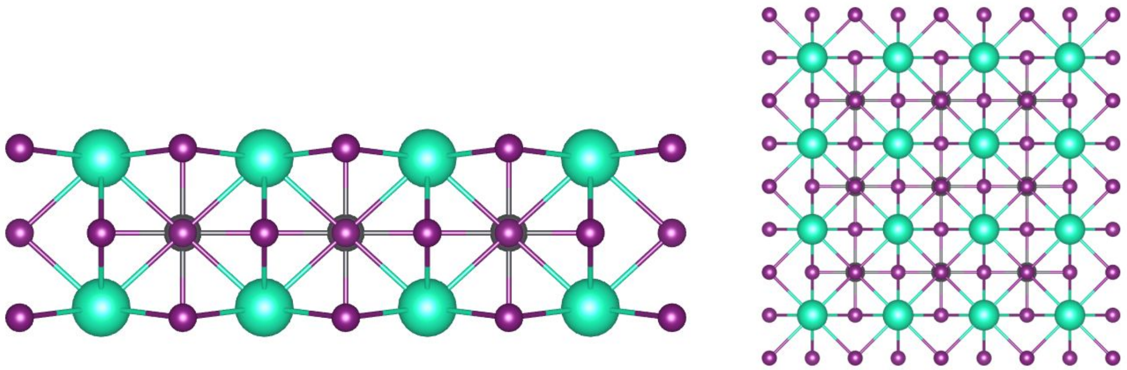

Figure S1. Lateral and top view of the PEA_2PbI_4 perovskite used in the simulations. The organic molecule is substituted by a cation atom for improving calculation efficiency. Cation atoms are shown as large green balls, Pb atoms as medium-sized purple balls, and I atoms as small purple balls.

Note 1. Determination of size of crystallites on the basis of the XRD lines broadening.

With use of Scherrer formula (S1), crystallite sizes D are determined for 5 XRD lines (Table S1).

$$D = \frac{K\lambda}{\beta \, cos\theta} \quad \text{(S1)}$$

where D is a crystallite size, K is the Scherrer constant (0.9), λ is the X-ray source wavelength (0.154406 nm), β is the FWHM (radians), θ is the peak position (radians)

Table S1. Data for crystallite size calculations.

Peak	Peak position (θ)	FWHM	Crystallite size (nm)	Average size (nm)
1	5.48218	0.07131	111.7830787	91.73356275
2	10.89191	0.06974	114.6864588	
3	16.33389	0.09057	88.81205003	
4	21.81625	0.09829	82.49708734	
5	27.35212	0.13458	60.88913887	

On the basis of the data presented we conclude that the crystallite sizes are between 60 and 120 nm.

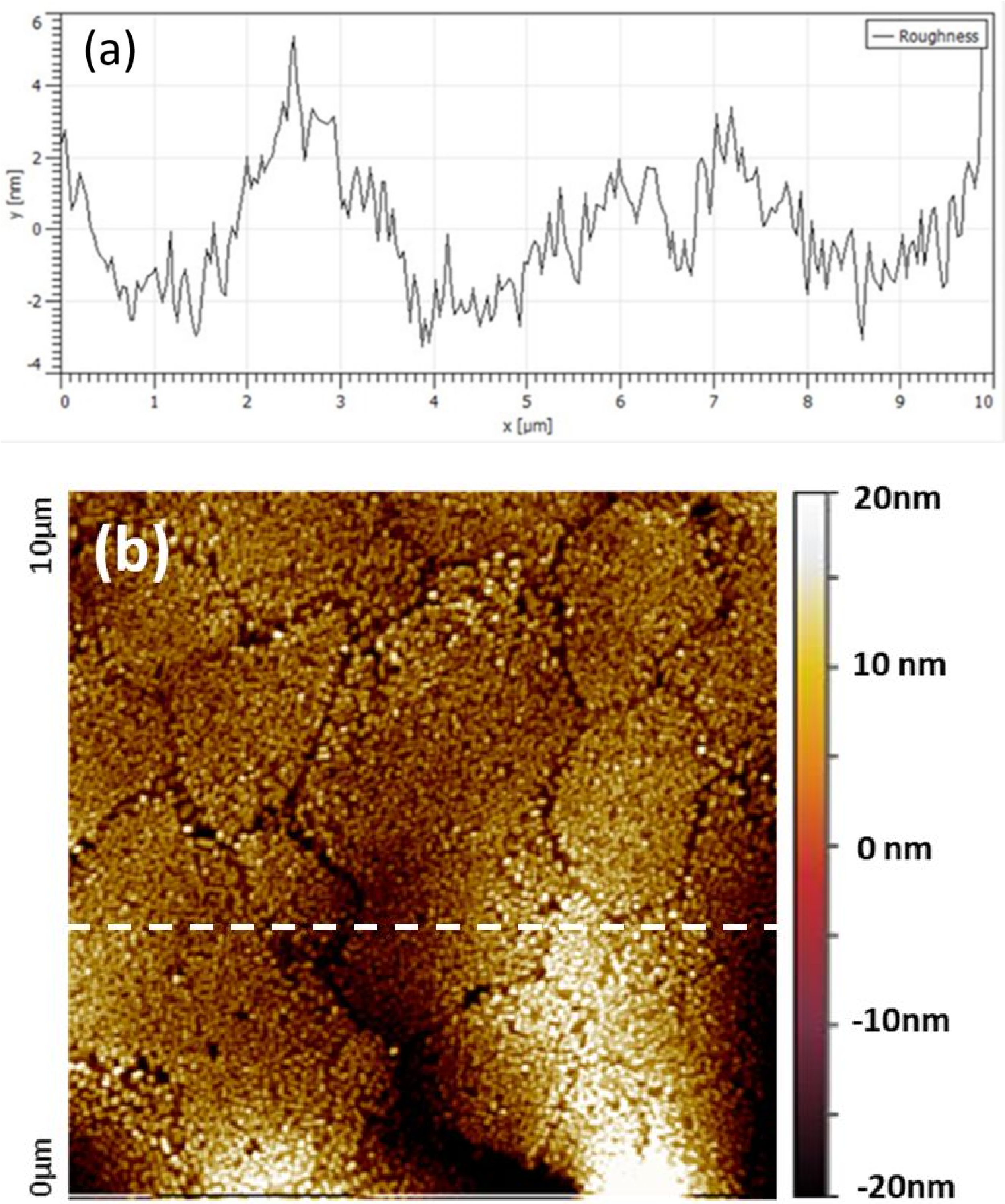

Figure S2. (a) Surface roughness profile of the AFM image shown in Fig. 1d and Fig. S2b. (b) AFM image of $(PEA)_2PbI_4$ polycrystalline film with an indicated line along which the above roughness profile was determined.

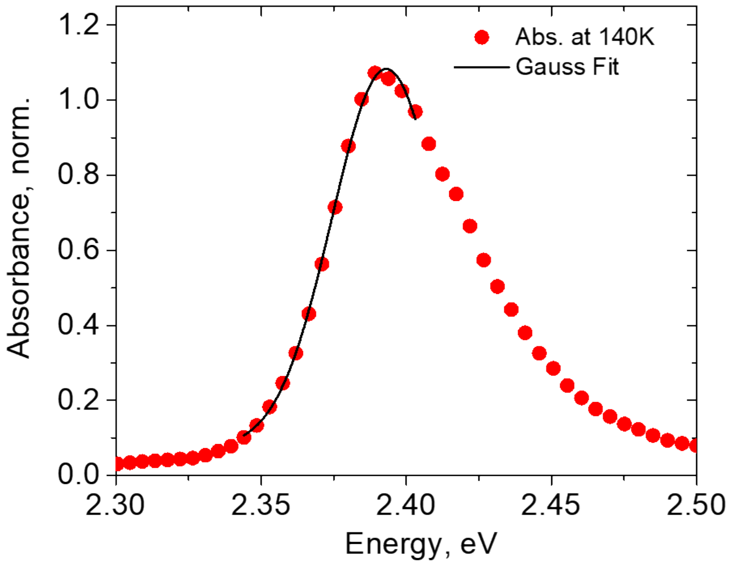

Figure S3. An example of fitting of the exciton absorption band of $(PEA)_2PbI_4$ polycrystalline layer by a Gaussian contour. Because of asymmetrical shape of the absorption band, the fitting was realized only for low-energy side and maximum of the experimental absorption band. The FWHMs of the Gaussian contours determined at different temperatures were used in Figure 2c.

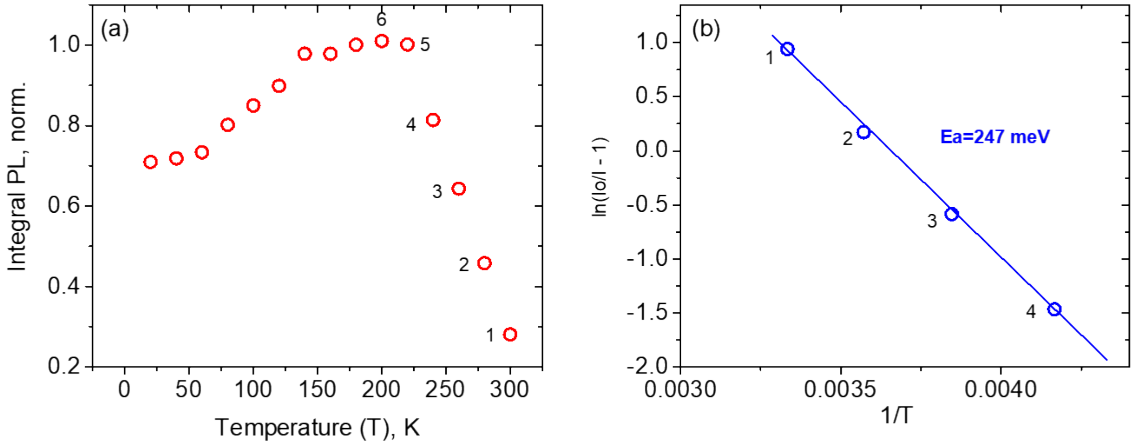

Figure S4. (a) Temperature dependence of the integral PL of $(PEA)_2PbI_4$ polycrystalline layer and (b) Arrhenius activation energy (E_a) determination. **Note:** The dependence of the integral PL intensity I on temperature T is considered to be described by the formula $I = {}^{I_0}/_{[1 + a \cdot \exp\left(-\frac{E_a}{kT}\right)]}$ where I_0 is the initial (unquenched) PL intensity, a is a constant, k is Boltzmann constant, E_a is Arrhenius activation energy. Linearization of *I(T)* dependence is realized in the coordinates

$\ln\left({}^{I_0}\!/_{I} - 1\right)$ vs ${}^{1}\!/_{T}$ where I_0 is the value of the point 6. The points 1-4 turn to lie on a straight line, a slope of which results in E_a = 247 meV.

REFERENCES

[1] Straus, D. B.; Parra, S. H.; Iotov, N.; Gebhardt, J.; Rappe, A. M.; Subotnik, J. E.; Kikkawa, J. M.; Kagan, C. R. Direct Observation of Electron-Phonon Coupling and Slow Vibrational Relaxation in Organic-Inorganic Hybrid Perovskites. *J. Am. Chem. Soc.* **2016**, *138*, 13798-13801.

[2] Sangalli, D.; Ferretti, A.; Miranda, H.; Attaccalite, C.; Marri, I.; Cannuccia, E.; Melo, P.; Marsili, M.; Paleari, F.; Marrazzo; Prandini, G.; Bonfà, P; Atambo, M. O.; Affinito, F.; Palummo, M.; Molina-Sánchez, A; Hogan, C.; Grüning, M.; Varsano, D.; Marini, A. A. Many-Body Perturbation Theory Calculations Using the Yambo Code. *J. Phys.: Condens. Matter* **2019**, *31*, 325902.

[3] Giannozzi, P.; Baroni, S.; Bonini, N.; Calandra, M.; Car, R.; Cavazzoni, C.; Ceresoli, D.; Chiarotti, G. L.; Cococcioni, M.; Dabo, I.; Corso, A. D.; de Gironcoli, S.; Fabris, S.; Fratesi, G.; Gebauer, R.; Gerstmann, U.; Gougoussis, C.; Kokalj, A.; Lazzeri, M.; Martin-Samos, L.; Marzari, N.; Mauri, F.; Mazzarello, R.; Paolini, S.; Pasquarello, A.; Paulatto, L.; Sbraccia, C.; Scandolo, S.; Sclauzero, G.; Seitsonen, A. P.; Smogunov, A.; Umari, P.; Wentzcovitch, R. M. QUANTUM ESPRESSO: a Modular and Open-Source Software Project for Quantum Simulations of Materials. *J. Phys. Condens. Matter* **2009**, *21*, 395502.
